# Epithelial to Mesenchymal Transition of a Primary Prostate Cell Line with Switches of Cell Adhesion Modules but without Malignant Transformation

**DOI:** 10.1371/journal.pone.0003368

**Published:** 2008-10-13

**Authors:** Xi-Song Ke, Yi Qu, Naomi Goldfinger, Kari Rostad, Randi Hovland, Lars A. Akslen, Varda Rotter, Anne Margrete Øyan, Karl-Henning Kalland

**Affiliations:** 1 The Gade Institute, University of Bergen, Bergen, Norway; 2 Department of Molecular Cell Biology, Weizmann Institute of Science, Rehovot, Israel; 3 Center of Medical Genetics and Molecular Medicine, Haukeland University Hospital, Bergen, Norway; 4 Department of Pathology, Haukeland University Hospital, Bergen, Norway; 5 Department of Microbiology and Immunology, Haukeland University Hospital, Bergen, Norway; Universität Heidelberg, Germany

## Abstract

**Background:**

Epithelial to mesenchymal transition (EMT) has been connected with cancer progression *in vivo* and the generation of more aggressive cancer cell lines *in vitro*. EMT has been induced in prostate cancer cell lines, but has previously not been shown in primary prostate cells. The role of EMT in malignant transformation has not been clarified.

**Methodology/Principal Findings:**

In a transformation experiment when selecting for cells with loss of contact inhibition, the immortalized prostate primary epithelial cell line, EP156T, was observed to undergo EMT accompanied by loss of contact inhibition after about 12 weeks in continuous culture. The changed new cells were named EPT1. EMT of EPT1 was characterized by striking morphological changes and increased invasion and migration compared with the original EP156T cells. Gene expression profiling showed extensively decreased epithelial markers and increased mesenchymal markers in EPT1 cells, as well as pronounced switches of gene expression modules involved in cell adhesion and attachment. Transformation assays showed that EPT1 cells were sensitive to serum or growth factor withdrawal. Most importantly, EPT1 cells were not able to grow in an anchorage-independent way in soft agar, which is considered a critical feature of malignant transformation.

**Conclusions/Significance:**

This work for the first time established an EMT model from primary prostate cells. The results show that EMT can be activated as a coordinated gene expression program in association with early steps of transformation. The model allows a clearer identification of the molecular mechanisms of EMT and its potential role in malignant transformation.

## Introduction

Epithelial to mesenchymal transition (EMT) is a developmental process characterized by loss of epithelial markers, gain of mesenchymal markers and changes in cellular morphology and phenotype with increased ability to migrate and invade [1]–[3]. EMT is an important physiological process during embryogenesis and wound healing, but may be exploited to play a central role in cancer progression (reviewed in [2], [4]–[7]). During progression to metastatic competence, carcinoma cells acquire mesenchymal adhesive properties and the activation of proteolysis and motility, which allows the tumor cells to metastasize and establish secondary tumors at distant sites [3]–[5]. Several models have been proposed to show that EMT contributes to the progress of established tumours in transformed cells [6], [8], [9]. The relationship between EMT and early carcinogenesis has not been clarified, and has so far been addressed by very few studies [10], [11].

Recently, it was described that EMT has strong and significant associations with multiple end points of prostate cancer progression and cancer-specific death [12]. It is therefore interesting to establish an EMT model based on prostate cells *in vitro*. EMT has been described in prostate cancer cells, including LNCaP [13], [14], DU145 [15] and PC3 cells [16]–[18]. However, multiple genomic rearrangements have accumulated in these cell lines during long term passages in different laboratories, and they are likely to differ significantly from the original patient cells. For PC3 cells, *e.g.*, both mesenchymal to epithelial transition (MET) [19] and EMT has been reported. More exact analysis of EMT will benefit much from cell lines that are very close to prostate cells *in vivo*.

EP156T is a primary prostate cell line immortalized by retroviral hTERT expression and retains many features of the prostate basal cell phenotype [20]. In an attempt to select for cells insensitive to contact inhibition [21], [22], EMT was observed in EP156T cells accompanied by loss of contact inhibition, and these changed new cells were named EPT1. The progeny EPT1 cells exhibited a distinct morphology and increased migration and invasion compared with the parental EP156T cells, and gene expression analysis revealed a striking number of gene expression changes previously found as markers of EMT [2], [9], [10], [23], [24]. Downregulation of E-cadherin (CDH1) and upregulation of N-cadherin (CDH2) - the so called cadherin-switch - were most evident in EPT1 cells. It appeared that entire gene modules involved in cell-to-cell and cell-to-matrix interactions changed their transcription patterns in a coordinated manner in EPT1 cells. Transformation assays revealed, however, that EPT1 cells still were serum and growth factor dependent and had not acquired ability to anchorage independent cell growth. This model therefore allows a study of EMT in primary prostate cells and separated from full malignant transformation.

## Results

### EP156T cells lost contact inhibition after long term culture at high confluence

The EP156T cell line is a primary prostate epithelial cell line immortalized by hTERT and has been previously characterized [20]. EP156T cells exhibit a significant pattern of authentic prostate-specific features with the capacity to differentiate into glandular buds that closely resemble the structures formed by primary prostate epithelial cells. It has previously been shown that ectopic expression of hTERT may be sufficient to immortalize cells *in vitro*, but not sufficient to achieve transformation [25]. This is consistent with the behaviour of EP156T cells, which exhibited properties of primary prostate basal cells [20]. EP156T cells grow in a monolayer and cease proliferation upon reaching confluence, a phenomenon referred to as cell contact inhibition.

**Figure 1:**
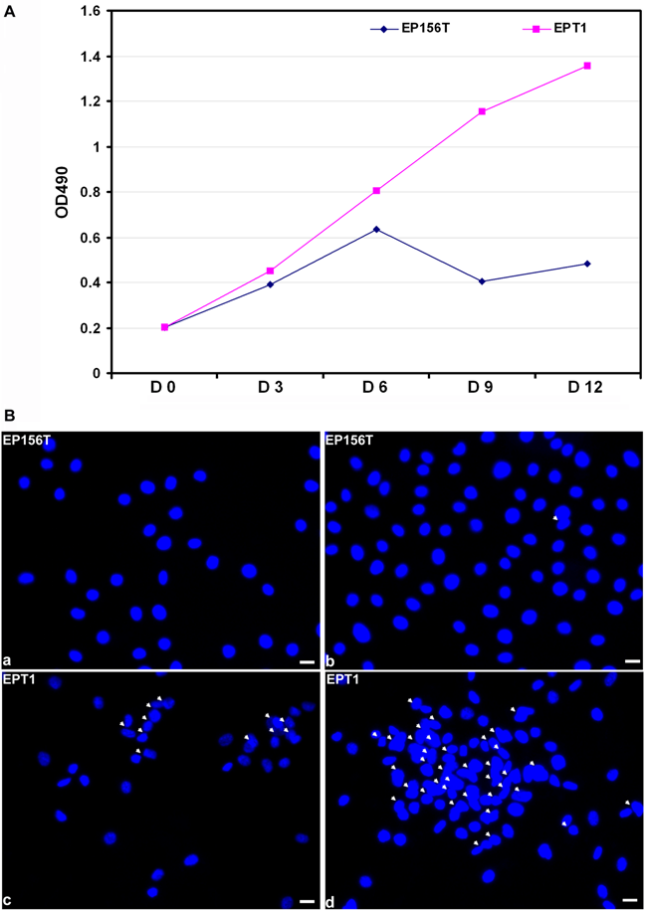
EPT1 cells have lost contact inhibition. A. Representative growth curves of EP156T and EPT1 cells. Both EP156T and EPT1 cells (3×10^3^) were seeded into 96-well plates and cultured in medium containing 1% FCS. Medium was changed every 3 days. At the indicated days (D) MTS reagent was added directly to culture wells and cell proliferation was calculated from the absorbance at 490 nm following incubation for 3 hours at 37°C. Data are presented as the average OD490 in three independent experiments. B. DAPI nuclear staining of EP156T and EPT1 cells at low density (a, c) and high density (b, d). White arrows indicate overlapping nuclei. White bars indicate 10 µm.

Loss of contact inhibition is one feature of malignant transformation [26]. One approach of transformation is to select cells that are insensitive to contact inhibition [22]. In order to transform EP156T cells to prostate cancer cells, the cells were kept growing at passage 43 at high confluence to select cells that survived contact inhibition. Cell death increased once they reached full confluence. Following 12 weeks thereafter with very slow proliferation, cells started to grow faster and kept on dividing and piled up despite high cell density. The changed cells were named EPT1. The growth curves of EPT1 and EP156T cells clearly displayed different behavior when cells were grown at high confluence. Many of the EP156T cells died while EPT1 cells continued proliferation (Figure 1A). Actually, EPT1 cells can grow on top of each other and pile up even under low cell density as shown in the DAPI nuclear staining in Figure 1B. Loss of cell contact inhibition may emerge before anchorage-independent cell growth and tumour formation in animals during the transformation process [27], [28]. The ability of EPT1 cells to override contact inhibition suggested development in the direction of transformation.

### EMT emerged during the change from EP156T to EPT1

**Figure 2:**
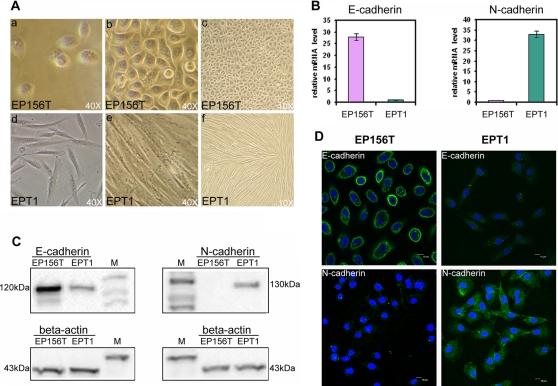
EPT1 cells have undergone EMT. A. Representative light microscopic images show morphological changes of EPT1 cells derived from EP156T cells at both low density (a, d) and high density cultures (b, c, e, f). EPT1 cells are fusiform and much longer than EP156T cells. Overlapping growth of EPT1 cells is also shown (d, e, f). B. Real-time qPCR showing the cadherin switch of mRNA levels in EPT1 compared to EP156T using TaqMan assays for E-cadherin (Hs00170423_m1) and N-cadherin (Hs00169953_m1). C. Western blots show the cadherin switch of protein levels. Both primary antibodies against E-cadherin and N-cadherin (BD Transduction Labs.) were diluted 1∶2500. The anti-actin mouse Mab was diluted 1∶250. The HRP-conjugated anti-Ig was diluted 1∶8000. Molecular weights (kD) were estimated based upon the ECL DualVue Marker in wells labelled M. D. Indirect immunofluorescence images (20×) show decreased E-cadherin and increased N-cadherin in EPT1 cells (right panels). The primary antibody against E-cadherin was diluted 1∶50 and against N-cadherin (BD Transduction Labs.) 1∶25 and the FITC-conjugated secondary antibody 1∶250.

Apart from their loss of contact inhibition, EPT1 cells changed significantly in morphological features compared with EP156T cells. EP156T cells have a very clear and round boundary with individual cells abutting on each other in a uniform array. There are regularly spaced cell to cell junctions and adhesions between neighbouring cells (Figure 2A). Very differently, EPT1 cells have a much longer and irregularly scattered cell shape, characteristically varying in composition and density like mesenchymal cells (Figure 2A) and corresponding to known features of epithelial to mesenchymal transition [2]. The morphological traits of EMT were consistent with key differences between EP156T and EPT1 cells regarding E-cadherin and N-cadherin gene expression as shown in Figure 2B–D and detailed below. hTERT was expressed at similar levels in both EP156T and EPT1 and the exogenous nature was supported by very high puromycin resistance of these cells, which is consistent with hTERT expression from a puromycin resistance retroviral vector (data not shown) [20]. EPT1 cells were trypsinized and propagated in standard EP156T medium with FCS increased to 5%.

### Increased migration and invasion of EPT1 cells

**Figure 3:**
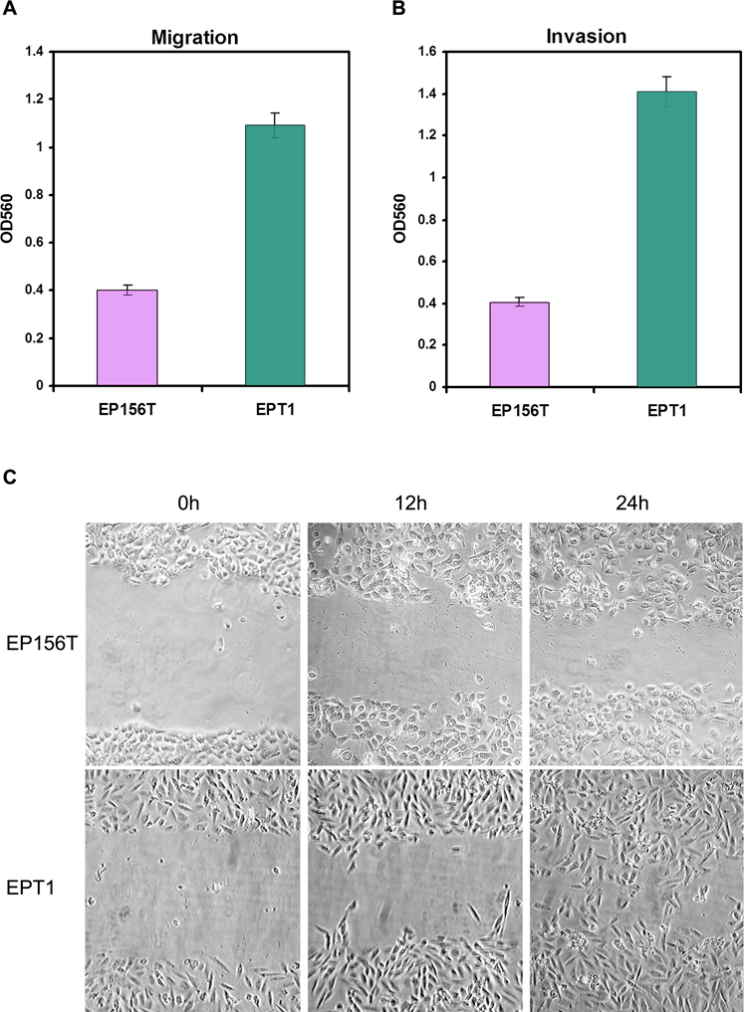
Migration and invasion of EP156T and EPT1 cells. The migration (A) and invasion (B) abilities of EP156T and EPT1 cells were examined as described in methods. Data are presented as the average OD560 of migrating cells (A) or invading cells (B) in four independent experiments. C. Representative light microscopic images (10×) show increased migration into a wound line of EPT1 compared to EP156T cells.

EMT is characterized by increased motility and gain of invasiveness. To determine whether EPT1 cells had become more active in migration and invasion than the EP156T cells, we evaluated the serum-induced migration of cells using transwell chamber assays. As shown in Figure 3A, EPT1 migrated through the pores more than 2.5 fold faster than EP156T cells. The invasive ability of EPT1 and EP156T cells was examined by the serum-induced invasion through the transwell extracellular matrix layer. As shown in Figure 3B, EPT1 cells exhibited 3 fold increase in invasion compared to EP156T cells after 24 h incubation. The faster migration of EPT1 compared with EP156T cells was also confirmed by the wound healing assay for 24 h (Figure 3C). EP156T cells moved as a *sheet en block* as described for epithelial cells, whereas EPT1 cells migrated considerably more dynamically and moved individually and sometimes left a part of the trailing region behind as described for mesenchymal cells [2]. All these results indicated that the EPT1 cells had acquired much higher ability to migrate and invade during the transition to mesenchymal-like cells in comparison with EP156T cells.

### EPT1 cells display striking gene expression switch patterns characteristic of EMT

To identify gene markers involved in EMT from EP156T to EPT1 cells, we profiled the gene expression of both cell lines using the Agilent Human Whole Genome Oligo Microarray, which contained 44 k probes. There were 965 genes downregulated and 893 genes upregulated more than 3 fold in EPT1 cells compared with EP156T cells. A number of gene expression changes associated with EMT varied significantly between EPT1 and EP156T (Table 1). Loss of E-cadherin (CDH1), the prototypic epithelial adhesion molecule in adherens junctions, and gain of N-cadherin (CDH2) are among the main hallmarks of EMT [2]. CDH1 was downregulated 27 fold and CDH2 upregulated 33 fold in EPT1 cells. The cadherin switch was verified by both real-time qPCR (mRNA level) and Western-blotting (protein level) (Figure 2B–C). Immunofluorescence staining showed very clearly the disappearance of E-cadherin and gain of N-cadherin in the membrane of EPT1 cells (Figure 2D). The epithelial markers including cytokeratin 14 (KRT14), KRT5 and p63 that characterized EP156T [20], were all downregulated more than 100 fold in EPT1 cells. In contrast, many mesenchymal markers were upregulated in EPT1 cells, including cadherin 11 (CDH11), vimentin (VIM) and fibronectin (FN). Apart from these EMT markers, several transcription factors that are known to regulate EMT, such as TWIST2 and ZEB1, were also upregulated in EPT1 cells (Table 1).

**Table 1:** Known markers of epithelial and mesenchymal cells were changed in EPT1 cells.

Genes	Fold change EPT1/EP156T	p-value	References
**Epithelial cell markers**			
CDH1	−28	3E-6	Kim JB [62]
CDH3	−138	2E-10	Jarrard DF [63]
DSP	−9	6E-5	Savagner P [64]
OCLN	−7	5E-3	Medici D [65]
KRT5	−538	4E-11	Kogan I [20]
KRT14	−32	2E-13	Kogan I [20]
**Mesenchymal cell markers**			
CDH2	33	1E-10	Lee JM [2]
CDH11	6	1E-14	Sarrió D [67]
FN1	4	7E-3	Yang Z [68]
VIM	3	13E-3	Billottet C [69]
FBN1	74	8E-15	Kiemer AK [23]
FGF1	3	2E-6	Billottet C [69]
FGFR1	9	1E-12	Acevedo VD [66]
SPARC	6	2E-2	Gotoh N [70]
**Known regulators of EMT**			
TWIST2	3	9E-5	Yang J [71]
WNT5A	4	1E-4	Dissanayake SK [72]
BMP4	3	1E-3	Theriault BL [73]
FZD7	3	1E-3	Vincan E [74]
ZEB1	3	10E-12	Eger A [75]

### Entire modules of genes encoding structural components of cell junctions and attachment were changed in EPT1

Cell junctions, especially adherens junctions, tight junctions and desmosomes, are required for the epithelial phenotype and keeping neighbouring epithelial cells strongly attached to each other [29]. The dynamic formation and dissolution of cell-cell junctional complexes is a central process during EMT [3]. Apart from the adherens junctions mentioned above, dissociated tight junctions [30], [31] or desmosomes [10] were reported as important features of EMT, respectively.

Using Agilent Whole Human Genome Microarray data, we compared the expression patterns of genes involved in adherens junctions, tight junctions and desmosomes between EP156T cells and EPT1 cells. As shown in Table 2, the majority of the examined components of these three groups were expressed at a much lower level in the EPT1 cells than in the parental EP156T cells (Table 2), such as E-cadherin, P-cadherin, β1 and δ1 catenins in adherens junctions, claudin 1, 4 and 7 in tight junctions, desmoglein 2 and 3 and desmoplakin 2 and 3 in desmosomes.

**Table 2:** Expression of cell junction genes in EPT1 cells.

Genes	Fold change EPT1/EP156T	p-value
**Desmosome**		
DSG2	−2	1.9E-3
DSG3	−93	7.0E-7
DSC2	−8	2.7E-7
DSC3	−6	7.7E-7
DSP	−9	1.5E-3
JUP	−18	2.9E-7
PKP1	−7	7.2E-6
PKP2	−6	4.0E-7
PKP3	−19	5.0E-9
PPL	−39	4.9E-5
EVPL	−2	1.0E-4
**Adherens junction**		
CDH1	−24	6.9E-8
CDH2	33	1.0E-8
CDH3	−120	6.5E-8
CTNNB1	−3	8.0E-4
CTNND1	−2	1.7E-3
Nectin	4	2.5E-6
**Gap junction**		
GJB2	−20	7.7E-7
GJB3	−87	7.3E-10
GJB4	−17	3.8E-7
GJB5	−36	6.0E-10
GJB6	−39	5.8E-6
**Tight junction**		
CLDN1	−3	2.5E-2
CLDN4	−2	9.6E-4
CLDN7	−20	1.2E-5
OCLN	−4	4.8E-3
**Hemidesmosome**		
DST	−37	5.1E-8
KRT4	−3	4.2E-2
KRT5	−538	3.5E-11
KRT 13	−12	1.2E-4
KRT 14	−32	2.2E-13
KRT 15	−44	8.6E-15
KRT 16	−42	3.3E-4
KRT 17	−177	2.0E-7
KRT 23	−41	5.0E-4
COL17A1	−78	3.2E-5
ITGB4	−11	2.5E-5
**Focal adhesion**		
CAV1	−3	9.8E-4
LAMA3	−11	4.3E-5
LAMA5	−4	5.0E-5
ITGB4	−11	2.5E-5
ITGB6	−6	2.6E-6
ITGB8	−5	1.0E-5
PFN1	−2	2.0E-3
PTK2	−2	1.7E-6
PTK2B	−5	4.1E-2
CFL2	6	3.3E-5
ENAH	2	2.4E-5
ITGA11	4	4.6E-9
LPXN	3	3.3E-5
PARVA	2	2.8E-3
TLN2	2	1.2E-5

Expression changes of cell junction genes in EPT1 cells according to Agilent Human Whole Genome Oligo Microarrays. Results were presented as fold change compared with EP156T cells. GAPDH expression is given as internal control. Three arrays were done for each cell type. Minus fold change represents underexpressed genes in EPT1 cells.

Very interestingly, it was also revealed that genes encoding other structural components of cell junctions were significantly downregulated in EPT1 compared to EP156T cells (Table 2). Gap junctions connect the cytoplasms of adjacent cells through the end-to-end docking of single-membrane structures. Most of the members of gap junction protein beta family exhibited dramatically reduced expression in EPT1 cells (Table 2). Hemidesmosomes and focal adhesions are required for epithelial cells to attach to the underlying basement membrane. Most components of the hemidesmosomes were downregulated in EPT1 cells compared with the parental cells, especially dystonin and keratins. Components of the focal adhesions were also changed in EPT1 cells (Table 2).

These observations, together with the consistently changed EMT markers, indicated that the regulation of EMT was orchestrated not only in cell phenotype transition, but also in entire modules of cell junctions. The complete changes of cell junctions make EPT1 an ideal model to study the complex regulatatory networks of EMT.

### EPT1 cells display gene expression patterns in common with prostate cancer cell lines

EMT has been frequently observed in transformed cell lines. We asked if EPT1 cells have similar gene expression profiles as prostate cancer cells represented by PC3 and DU145. Differentially expressed genes between EP156T and EPT1 and between EP156T and prostate cancer cell lines were compared. In total 1858 genes differed more than 3 fold between EPT1 and EP156T cells. As shown in Figure S1, most of the genes altered after EMT in EPT1 cells were also altered when comparing the PC3 and DU145 to the EP156T cells. More than 70% of the downregulated genes in EPT1 overlapped with genes underexpressed in PC3 cells compared to the EP156T cells. Interestingly, in PC3 cells many epithelial markers including E-cadherin and the cytokeratins KRT4 and KRT5 were also significantly downregulated, and the mesenchymal markers including N-cadherin were also upregulated. This is consistent with a previous report that PC3 cells are mesenchymal-like cells that can be induced to mesenchymal to epithelial transition (MET) by overexpression of secreted Frizzled-related protein 3 (sFRP3) [19]. Both the loss of contact inhibition of EPT1 and the similar gene expression changes of EPT1 and prostate cancer cells versus EP156T cells indicated a development towards transformation of EPT1 cells.

### EPT1 cells are not transformed although they have undergone EMT

**Figure 4:**
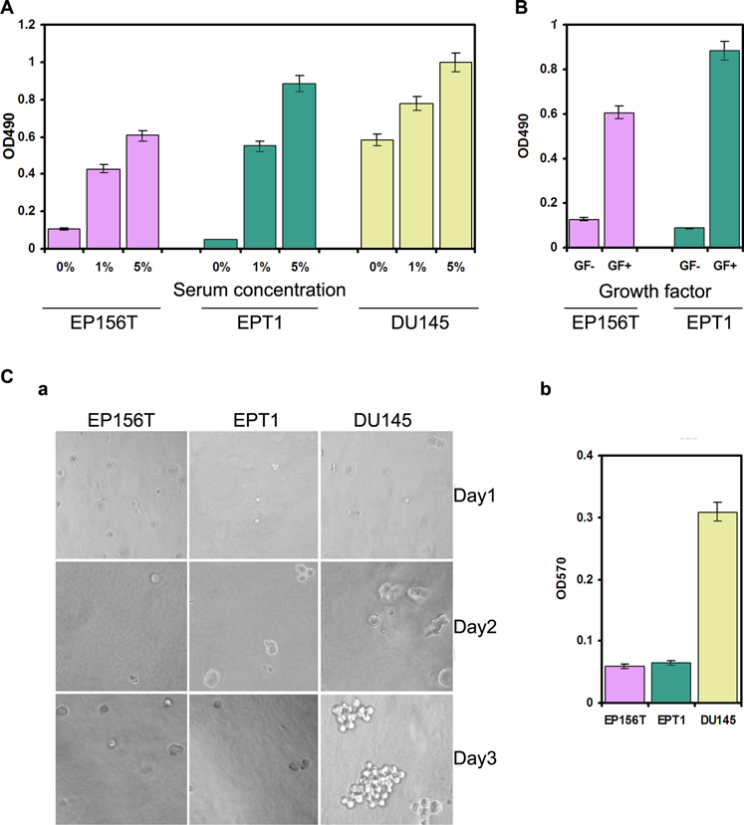
Transformation assays of EPT1 cells. A. EP156T, EPT1 and DU145 cells were cultured in medium containing 0%, 1% and 5% FCS, respectively. B. EP156T and EPT1 cells were cultured in the absence (GF−) or presence (GF+) of growth factors in the medium. C. Assay of anchorage independent growth a. Representative images (10×) of colony formation in soft agar at different times as indicated. During the first 3 days, some EPT1 cells kept dividing while EP156T did not. Neither EP156T nor EPT1 cells could grow in soft agar eventually, while many colonies formed in prostate cancer DU145 cells. b. Following 7 days incubation in soft agar cells were quantified as absorbance at OD570 nm as described in methods.

The observation that changed genes in EPT1 share patterns with prostate cancer cell lines made us ask whether EPT1 cells were transformed. An ability to survive and proliferate under serum-free condition is one of the well-known features of cancer cells *in vitro* [32]. We examined the proliferation of EPT1 cells in medium with different concentrations of fetal calf serum (FCS). As shown in Figure 4A, both EP156T and EPT1 cells grew much slower in 1% FCS than in 5% FCS and completely stopped growing and died in medium without FCS after 48h incubation, while prostate DU145 cancer cells still grew well in FCS-free medium although slower than in complete medium. The long term culture showed that EPT1 cells not only grew faster, but also looked more vigorous in medium containing 5% FCS than in the standard EP156T medium, so the concentration of FCS in the medium for EPT1 cells was adjusted from 1% to 5%.

One of the most important capabilities of cancer cells is self-sufficiency in growth signals [33]. The complete medium of EP156T and EPT1 cells contained many kinds of growth factors including bovine pituitary extract, insulin and EGF [25]. When the EP156T and EPT1 cells were cultured in basic medium without the above growth factors, most of the cells in both cell lines stopped to proliferate and died after 48 h, while the parallel cells in complete medium grew well (Figure 4B).

Anchorage-independent growth is considered a hallmark of transformed cells [34], [35]. We tested anchorage-independent growth of EPT1 cells in soft agar. Following 7 days incubation in soft agar, neither EP156T nor EPT1 cells were able to form colonies in contrast to positive control DU145 cells (Figure 4). Although neither formed colonies in soft agar, a difference was observed. Nearly half of EPT1 cells divided during the first 3 days, but then proliferation stopped without colony formation in the end. In contrast, EP156T cells did not divide at all in soft agar. These differences in soft agar growth suggested that EPT1 cells are in a stage of premalignancy following EMT (Figure 4). EMT and transformation assays of EPT1 are summarized in Table 3.

**Table 3:** Summary of EMT and transformation assays of EPT1 cells.

EMT	Yes or No
***In vitro*** ** functional markers**	
Elongation of cell shape	Yes
Increased scattering	Yes
Increased migration	Yes
Increased invasion	Yes
**Increased mesenchymal markers**	
N-cadherin/Vimentin/Fibronectin/Integrin	Yes
**Decreased epithelial markers**	
E-cadherin/Desmoplakin/Cytokeratin/Occludin	Yes

Transformation	Yes or No
Loss of contact inhibition	Yes
Chromosomal instability	No
Serum independent growth	No
Self-sufficiency in growth signals	No
Anchorage independent growth	No

### Cytogenetic analysis of EP156T and EPT1

Abnormal karyotypes are typically observed in transformed prostate epithelial cells [36]–[38]. By G-banding we found no pronounced chromosomal alterations occurring between EP156T and EPT1 cells (Figure S2). Most likely a clonal selection has occurred as the EPT1 cells did not show any of the additional marker chromosomes. Both the EP156T cells and the progeny EPT1 cells harboured a marker chromosome which by FISH (fluorescent *in situ* hybridization) analysis was shown to be a derivative chromosome 20 (der(20), Supplementary Figure 2), Whether gain of der(20) occurred prior to immortalization or as a consequence of EP156T immortalization is unknown. However, a gain of chromosome 20 was found in both non-malignant and malignant cells immortalised by hTERT [39]. As this gain is not a common finding in prostate cancer [40]–[42] this could result from the immortalization process.

## Discussion

This work is the first report of an EMT model based on primary, immortalized prostate epithelial cells. The derivation of EPT1 from EP156T was clearly verified by the similar expression levels of hTERT, the puromycin resistance and the very similar karyotypes of both cell lines, including a common derivative chromosome, der(20). Several ways to induce EMT in prostate cancer cells have been described, including overexpression of CAV1 and ID1 or MMP14 in LNCaP cells [13], [14], EGF treatment of DU145 cells [15], depletion of PDEF [16] or BMP7 treatment of PC3 cells [18]. However, some of these studies cannot easily be reconciled, such as both EMT and MET induction of PC3 cells [16]–[19]. Long term passages of these cell lines in different laboratories may cause them to differ significantly from the original patient cells, and new models closer to prostate tissue are desirable.

EMT is not easily observed in histological examinations of cancer tissue sections, even by experienced pathologists [2], [43]. This has led to the idea that EMT may be transient during cancer progression [44], [45] or occur only in a subpopulation of the tumour cells, such as cells at the invasive front [5], [8], [46]–[48] or cancer stem cells [11], [49], [50]. The successful establishment of an EMT model based on primary prostate cells with many traits of the prostate basal cell phenotype is important in light of a recent report on the significance of EMT in prostate cancer[12].

EMT has been considered an event following malignant transformation to endow cancer cells invasive and metastatic competence [6], [8], [9]. The present work shows that primary prostate epithelial cells underwent complete EMT, while most of the transformation assays remained negative, including serum and growth factor independent growth, as well as anchorage independent growth and chromosome instability (Figure 4). At the same time, loss of contact inhibition combined with similar differential gene expression patterns as in prostate cancer cell lines indicated that EPT1 cells are in the early steps towards transformation. Regarding the temporal relationship between transformation and EMT, one recent report has also observed that EMT emerged at a very early stage of transformation (early HF1 cells) [10]. However, EMT in that system was far from full completion, lacking mesenchymal characteristics in both early and later transformation stages, and the downregulation of E-cadherin was significant neither in early (0.9±0.1) nor in later (0.5±0.2) stages of transformation, and N-cadherin was not found upregulated. To our knowledge, EPT1 cells represent the first model in which EMT has finished completely at very early stages of malignant transformation.

It has previously been shown that ectopic expression of hTERT may be sufficient to immortalize cells *in vitro*, but not sufficient to achieve, anchorage-independent cell growth or tumorigenesis [25]. Full malignant transformation is a stepwise process and can be achieved by additional expression of oncogenes such as the SV40 early region plus activated RAS [25], [51]. EMT was induced in the hTERT immortalized primary prostate epithelial EP156T cells without exogenous introduction of oncogenes and without transformation. Furthermore, karyotype analysis revealed that the EPT1 cells were diploid and apparently contained much less karyotypic abnormalities than previously described for the prostate cancer cell lines LNCaP, PC3 or DU145 [52], [53]. This work shows that EMT itself appears as a coordinated gene expression program that may be switched on in either non-transformed or transformed cells. It is possible that EMT may contribute towards malignant transformation in non-transformed cells, and that in transformed cells EMT may contribute to a more aggressive phenotype. Genomic features of transformation such as loss of heterozygocity or chromosomal rearrangements cannot easily be reversed. In this respect EMT regulation may provide an important target for cancer therapy. Work is ongoing to further transform EPT1 cells by additional stimulation or oncogene introduction. This model is therefore valuable for understanding the mechanistic relationship between EMT and transformation.

EPT1 cells lost contact inhibition accompanied by EMT during the selection of cells insensitive to full confluence. EP156T cells ceased growing once cells abutted on each other in contrast to EPT1 cells whose protrusions passed across neighbouring cells, eventually leading to heaps of cells. It is still unknown what happened first or emerged simultaneously in EPT1 cells. Actually, both contact inhibition and increased invasion and migration in EMT are associated with cell to cell junctions [54]. The gene expression of entire modules of cell junctions such as adherens junctions, gap junctions, tight junctions, desmosomes and hemidesmosomes were downregulated almost simultaneously as EPT1 arose from EP156T cells. These cell junction modules not only can contribute to the interaction of epithelial cells and provide a barrier against increased migration and invasion in EMT[2], but also can lead to cell contact inhibition based on cell communication [55]. It has been shown that adherens junctions play an important role in contact inhibition of cell growth [56] and components of gap junctions induce cell contact growth inhibition [57]. One recent publication showed that EMT mediated loss of contact inhibition in hepatocarcinoma cells, and reexpression of E-cadherin restored cell-cell contacts [58]. Work is ongoing to identify the key regulatory mechanisms behind the observed switches of entire functional gene expression modules in EPT1 during EMT and the relationship between loss of contact inhibition and EMT in EPT1 cells.

## Materials and Methods

### Antibodies, cell lines and cell culture

Mouse monoclonal antibodies were used to detect E-cadherin (BD Transduction Labs., BD Biosciences, San Jose, CA USA, Cat#610181), N-cadherin (BD Transduction Labs. Cat# 610920 and Abcam, Cambrigde, GB, Cat.#ab19348) and β-actin (Abcam Cat# ab11003). The prostate cancer cell lines DU145 and PC3 were obtained from the American Type Culture Collection (ATCC, Rockwell, MD, USA). The derivation and growth conditions for the EP156T cell line have been described [20]. Additional reagents were from Sigma-Aldrich, St. Louis, MO, USA unless indicated otherwise.

### Generation of EPT1 cells

EP156T cells were allowed to grow to full confluence at passage 43 in 6 well plates in EP156T standard medium essentially as described [20]. This medium was modified MCDB153 (Biological Ind. Ltd, Israel) and was supplemented with 1% MEM non-essential amino acids solution, 200 nM hydrocortisone, 10 nM triiodothyronine, 5 µg/ml insulin, 5 µg/ml transferrin, 5 µg/ml sodium selenite, 100 ng/ml testosterone, 5 ng/mL EGF, 50 µg/mL bovine pituitary extract (Invitrogen), 100 U/ml penicillin, 100 µg/ml streptomycin and 1% fetal calf serum (FCS). The medium was changed every 3 days. Twelve weeks later cells were trypsinated and transferred to new plates and grown in EP156T medium with FCS increased to 5%.

### Proliferation, migration, invasion and wound closure assay

For the proliferation assay, 3500 cells in complete medium were added to each well of the 96-well plate. After time course incubation, cell proliferation was assayed using the CellTiter96®AQ_ueous_ One Solution Cell Proliferation Assay (MTS) (Promega, Madison, WI, USA). MTS reagent was added directly to culture wells, and following incubation for 3 h at 37°C absorbance was recorded at 490 nm using a 96-well plate reader (Powerwave spectrophotometer). For trans-well migration assays and invasion assays, 1.8×10^5^ overnight serum starved cells in serum-free medium were added to the top chambers of 24-well trans-well plates (8 µm size, Cell Biolabs Inc., San Diego, CA, USA) and media containing 10% fetal calf serum (FCS) were added to the bottom chambers. Incubation was for 12 h in the migration assay and 24 h in the invasion assay. Top (nonmigrating) cells were removed, bottom (migrating) cells were stained and absorbance was recorded at 560 nm. All of these assays were done in triplicate and the data are presented as the average absorbance of migrating cells and invading cells.

For the scratch wound closure assay, 5×10^5^ cells were seeded in 6-wells. A wound was incised 24 h later in the high density area, detached cells were removed and fresh medium was added. Photos were taken of the wounded area at indicated time points.

### Microarray analysis and real-time quantitative PCR

Total RNA was extracted from subconfluent monolayers of cells. One µg of DNAse-treated total RNA was converted into cDNA and next to Cy3-labeled cRNA using the Low RNA Input Linear Amplification Kit PLUS, One-Color kit (Agilent Tech., Santa Clara, CA, USA), according to instructions. The Agilent Human Whole Genome (4×44 k) Oligo Microarray with Sure Print Technology (Agilent Technologies, Inc., Palo Alto, CA) was used to analyze samples in the present study. The hybridization and features extraction were described in [59]. Significant Analysis of Microarray (SAM) using the significantly up- or downregulated genes in EPT1 cells were performed in J-Express (www.molmine.com) [60]. Annotated microarray data were uploaded in the BASE database and formatted and exported to ArrayExpress at the European Bioinformatics Institute (http://www.ebi.ac.uk/arrayexpress (Accession number: E-BASE-8)) in agreement with the MIAME guidelines. Differentially expressed genes in microarray data were confirmed by real-time quantitative reverse transcription PCR (qPCR) using TaqMan assays (Applied Biosystems, Foster City, CA, USA) and mean quantitative values were normalized according to ACTB expression as described [59].

### Indirect immunofluorescence and Western blot assays

Indirect immunofluorescence (IF) and western blot (WB) analyses were done essentially as previously described [61]. For IF cells were grown on 10 mm Assistant glass coverslips in 24 well plates, then washed with PBS, fixed (4% fresh paraformaldehyde in PBS for 20 min. at room temperature), permeabilized (0.5% Triton X-100 for 10 min.), blocked (100 mM glycin for 10 min) and stored (100% methanol at 4°C) with PBS wash between each step. Following blocking with 0.5% BSA/PBS for 15 min. primary mouse monoclonal antibodies were added overnight at 4°C at indicated dilutions in 0.5% BSA/PBS. The FITC-labelled secondary anti-mouse Ig (SouthernBiotech, Birmingham, AL, USA, Cat# 4050-02) was added for 1 h at room temperature in 0.5% BSA/PBS. Coverslips were mounted in SlowFade with DAPI (Molecular Probes, Invitrogen, Carlsbad, CA, USA) on glass slides and analysed using Leica confocal microscopy.

For WB analysis cells were lysed in 200 mM Tris.Cl pH 6.8, 13% ultrapure glycerol, 3.2% β-mercaptoethanol, 40 g/l SDS and Protease Inhibitor Cocktail Set I diluted 1∶100 (Calbiochem, Cat# 535142). Protein concentrations were measured using the BCA protein Assay kit (Pierce, Rockford, IL, USA, Cat# 23227), and 20 µg protein lysates were separated by 10% polyacrylamide gel (Biorad Labs., Hercules, CA, USA, Cat#161-1173) SDS electrophoresis followed by blotting to PVDF membranes (GE Healthcare Life Sciences, Uppsala, Sweden, Cat# RPN1416F). Primary mouse monoclonal antibodies and HRP-labelled secondary antibodies (GE Healthcare, Cat.# NA931) were used at the indicated concentrations and the bands detected using enhanced chemiluminescence (GE Healthcare, Cat# RPN2132) and the Biorax Fluor-s multiImager. Molecular weight markers used were ECL DualVue Marker (GE Healthcare, RPN810) and MagicMark XP Western Protein Standards (Invitrogen, Cat# LC5602).

### Serum independent, growth factors independent assays

For the serum independent assay, cells were cultured in medium with 0%, 1% and 5% FCS, respectively. For the growth factors independent assay, cells were cultured in basic MCDB medium and complete medium, respectively [20]. The proliferation of each group was measured 48 h later as described above.

### Anchorage-independent growth assay

The anchorage independent growth was examined in soft agar. 50 µl of base agar matrix (Cell Biolabs; CytoSelect™ Cell, Colorimetric kit) was added in the bottom of each well of a 96-well plate. When the agar was solid, 75 µl of cell suspension/soft agar matrix containing 3000 cells was layered on top followed by 50 µl of 2× complete medium. After 7 days of incubation, the agar matrix was solubilized and cells stained and absorbance was recorded at 570 nm. Data show the quantification of proliferation of EPT1 cells, EP156T cells and positive control DU145 cells in the soft agar assay.

### Cytogenetic analysis

Prior to harvesting, the cells where treated with 20 ng/ml colcemid for 25 min at 37°C followed by trypsination. The cell suspension was washed once before exposing the cells to prewarmed (37°C) hypotonic solution (0.22% (v/v) NaCl, 0.3 mM KH_2_PO_4_, 80 mM Na_2_HPO_4_, pH 8.0). Cells were pelleted and re-suspended in 4 ml fresh fixative (3∶1 methanol∶acetic acid) which was repeated four times before storing the cell suspension at −20°C. G-banding was performed by the standard Giemsa staining procedure and metaphase spreads were analysed. The karyotype was described according to ISCN 2005. FISH (fluorescent *in situ* hybridization) analyses using probes (CEN20, TEL12q, LSI MLL) from Vvsis (Abbott Molecular–Vysis, Des Plaines, IL; Abbott Diagnostics, Maidenhead, UK) were performed according to the manufacturer's instruction.

## Supporting Information

Figure S1 Hierarchical clustering analysis of differentially expressed genes among DU145, PC3, EP156T and EPT1 cells. In total 1858 genes differed more than 3 fold between EPT1 and EP156T cells. The red and the blue represent low expression and high expression, respectively.
(4.20 MB TIF)

Figure S2 G-banding of metaphases of the EP156T cells (A) showed few chromosomal aberrations compared to prostate cancer cell lines. A marker chromosome was found in all cells (der(20), red arrow), but diverging clonal evolution as often seen in cell lines was also seen in the EP156T cells. One subclone had trisomy 13 (blue arrow), another loss of chromosome 8 and 20 and gain of chromosome 2, whereas others showed different marker chromosomes. The composite karyotype can be described as 46–48,XY,+2[2],−8[3],+13[4],−20[2],+der(20)[10],+mar[3][cp10]. G-banding analysis of the EPT1 cells (B) showed the same marker chromosome in all as found in the EPT156 cells (red arrow) but in addition a loss of the normal chromosome 20 (black arrow). Clonal evolution involving chromosome 13 (blue arrow) was also found. The composite karyotype can be described as: 46–47,XY,+13[3],+i(13)(q10)[2],der(20)[10],−21[3][cp10]. In order to gain more information on the source of the marker chromosome FISH analysis (C) was performed. A probe against centromer 20 revealed that the marker chromosome was a chromosome 20 derivative (der(20), red arrow), whereas a probe against the subtelomeric region of 12q showed no involvement of that chromosome.
(2.76 MB TIF)
