# Supplementary figures and images for: Epithelial to Mesenchymal Transition of a Primary Prostate Cell Line with Switches of Cell Adhesion Modules but without Malignant Transformation
Source: PLoS One. 2008 Oct 13;3(10):e3368. doi: 10.1371/journal.pone.0003368 (PMC2557125; doi:10.1371/journal.pone.0003368)

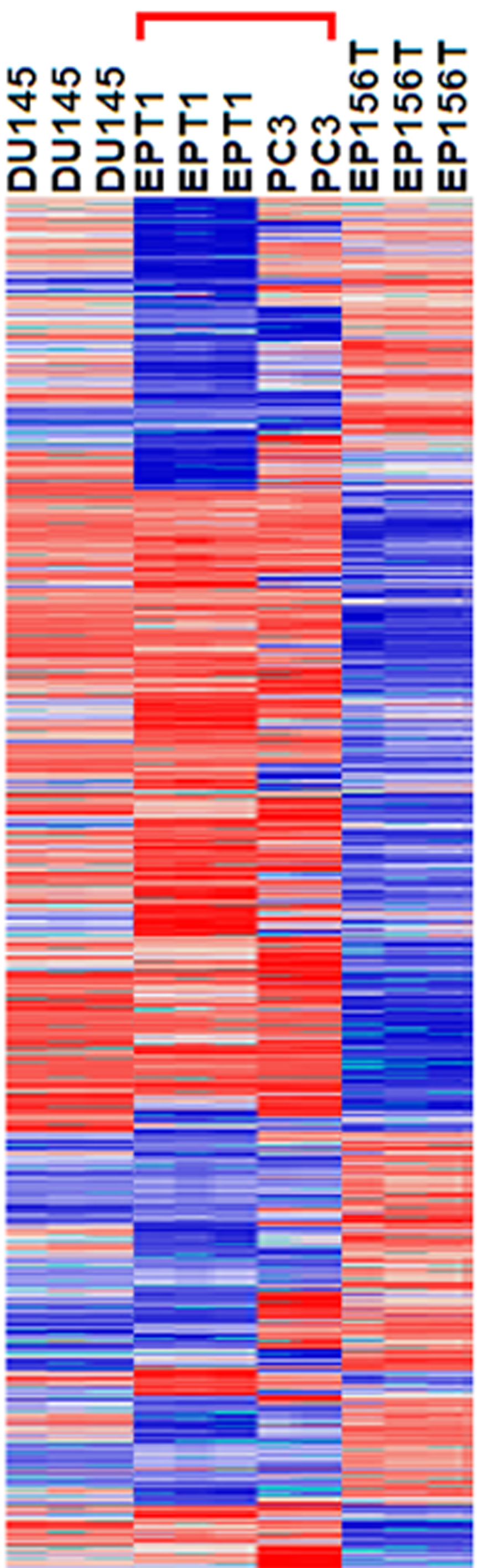

Supplement: Figure S1 — Hierarchical clustering analysis of differentially expressed genes among DU145, PC3, EP156T and EPT1 cells. In total 1858 genes differed more than 3 fold between EPT1 and EP156T cells. The red and the blue represent low expression and high expression, respectively. (4.20 MB TIF) [file pone.0003368.s001.tif]

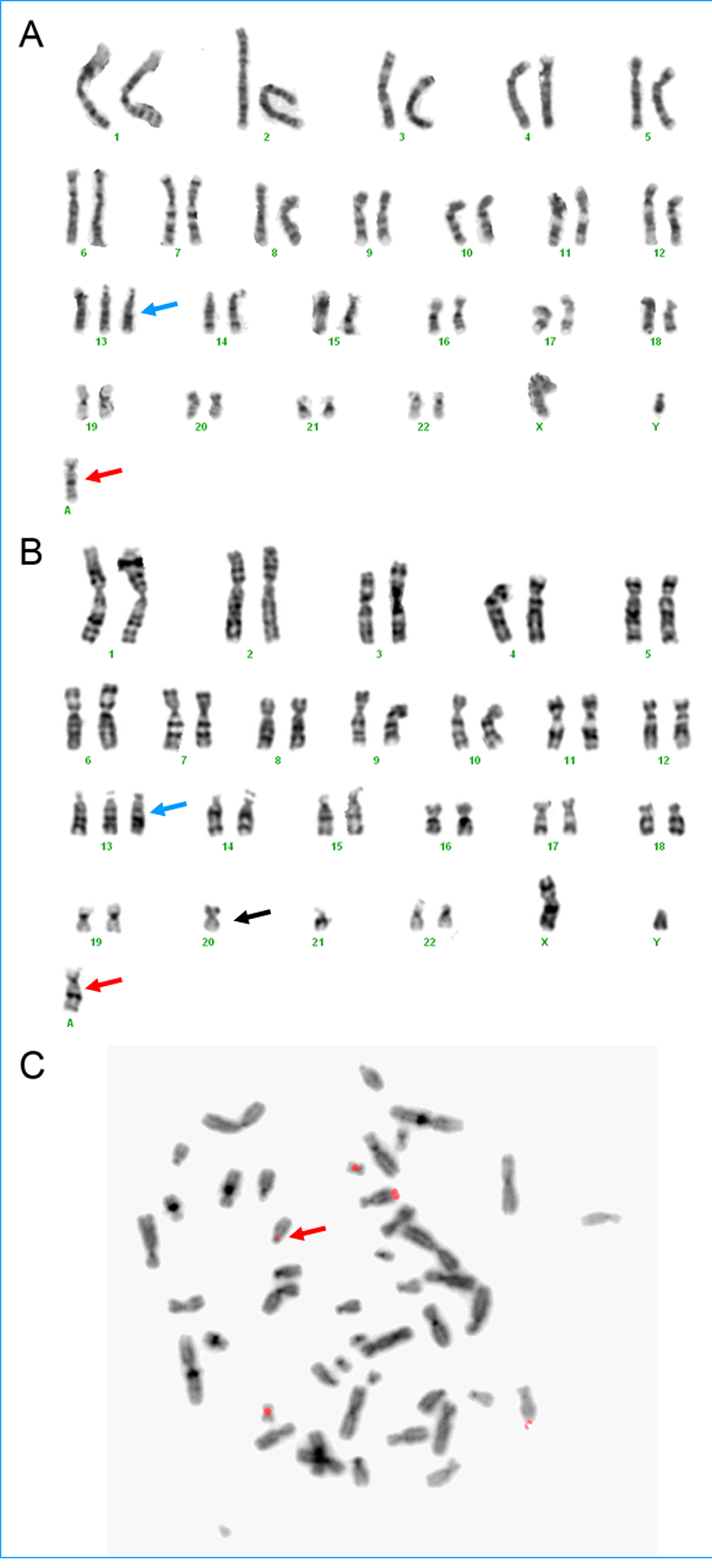

Supplement: Figure S2 — G-banding of metaphases of the EP156T cells (A) showed few chromosomal aberrations compared to prostate cancer cell lines. A marker chromosome was found in all cells (der(20), red arrow), but diverging clonal evolution as often seen in cell lines was also seen in the EP156T cells. One subclone had trisomy 13 (blue arrow), another loss of chromosome 8 and 20 and gain of chromosome 2, whereas others showed different marker chromosomes. The composite karyotype can be described as 46–48,XY,+2[2],−8[3],+13[4],−20[2],+der(20)[10],+mar[3][cp10]. G-banding analysis of the EPT1 cells (B) showed the same marker chromosome in all as found in the EPT156 cells (red arrow) but in addition a loss of the normal chromosome 20 (black arrow). Clonal evolution involving chromosome 13 (blue arrow) was also found. The composite karyotype can be described as: 46–47,XY,+13[3],+i(13)(q10)[2],der(20)[10],−21[3][cp10]. In order to gain more information on the source of the marker chromosome FISH analysis (C) was performed. A probe against centromer 20 revealed that the marker chromosome was a chromosome 20 derivative (der(20), red arrow), whereas a probe against the subtelomeric region of 12q showed no involvement of that chromosome. (2.76 MB TIF) [file pone.0003368.s002.tif]
